# Migratory Arthritis and Heart Block in an Adult: A Forgotten Diagnosis Revisited

**DOI:** 10.7759/cureus.90346

**Published:** 2025-08-17

**Authors:** Marius Dsouza, Boon J San, Suraj Adhikari

**Affiliations:** 1 Internal Medicine, Albert Einstein College of Medicine, Jacobi Medical Center, New York, USA

**Keywords:** acute rheumatic fever, adult onset acute rheumatic fever, migratory arthritis, revised jones criteria, subclinical carditis

## Abstract

Acute rheumatic fever (ARF) is an abnormal immunologic response to group A streptococcus (GAS) infections, most commonly tonsillopharyngitis. While ARF most often occurs in children, it is rare in adults. Carditis is a recognized manifestation of ARF, though it is uncommon in adult presentations. We present a case of a 35-year-old male with fever, migratory polyarthritis, and subclinical carditis in the form of a prolonged PR interval, mild aortic regurgitation (AR), and trace mitral regurgitation (MR) detected on echocardiography. Further investigations revealed an erythrocyte sedimentation rate (ESR) of 101 mm/hour and markedly elevated antistreptolysin O (ASO) titers of 1,600 IU/mL. The diagnosis of ARF was made based on the 2015 revised Jones criteria, which included one major criterion (migratory polyarthritis), three minor criteria (fever, elevated ESR, and prolonged PR interval), and supportive evidence of recent streptococcal infection. Other causes of arthritis, such as septic arthritis, were ruled out. The patient responded well to nonsteroidal anti-inflammatory drugs (NSAIDs) and was treated with intramuscular penicillin G for eradication, followed by lifelong azithromycin prophylaxis for recurrence prevention. This case highlights the importance of considering ARF in adult patients presenting with migratory arthritis and conduction abnormalities.

## Introduction

Acute rheumatic fever (ARF) is an immune-mediated, nonsuppurative complication of group A streptococcus (GAS) infection [1]. ARF can occur at any age, although most cases are seen in children between five and 15 years of age [2]. Household overcrowding, poor sanitation, and inadequate access to healthcare are significant contributors to the spread of GAS pharyngitis [3]. A major consequence of ARF is irreversible cardiac valve damage due to recurrent episodes, leading to rheumatic heart disease (RHD) [4]. Attacks of ARF in adults are uncommon and may represent either an initial episode or a recurrence after a childhood infection. Diagnosing ARF in adults presenting with arthritis without a prior history of ARF can be challenging, often leading to delays in diagnosis and treatment.

This article was previously presented as a poster at the Milford Fulop Poster Competition on May 15, 2025, at New York Health and Hospitals, Jacobi Medical Center.

## Case presentation

A 35-year-old male with a history of right sixth cranial nerve palsy nine years ago, presumed secondary to Lyme disease based on positive Lyme antibody testing at that time, presented with a two-week history of migratory polyarthritis. His initial symptoms began with left knee pain, mild swelling, and fever (101.8°F). He underwent synovial fluid analysis at another hospital for suspected septic arthritis but left against medical advice. Two weeks later, he presented to our center with right knee pain for one week, followed by pain in the left elbow, wrist, and first carpometacarpal joint for two days. The left knee pain had improved but persisted. All joint pain was exacerbated by movement and weight-bearing. He denied any recent sore throat, rash, palpitations, shortness of breath, focal weakness, or paresthesia.

On examination, his pulse was 90 beats per minute and regular. Blood pressure was 155/96 mmHg, temperature 98.4°F, respiratory rate 18 breaths per minute, and oxygen saturation 96% on room air. He was alert and oriented to time, place, and person. Oropharyngeal examination showed no erythema or exudate. Joint examination revealed mild suprapatellar swelling in both knees without erythema or warmth. Range of motion was intact but painful on movement and weight bearing. The left elbow showed marked tenderness on movement without swelling or warmth. The left wrist showed mild swelling and tenderness on movement without erythema. The left first carpometacarpal joint was tender to palpation and movement. Cardiac examination revealed normal heart sounds without murmurs or gallops. Lung auscultation was clear bilaterally. Abdominal examination was unremarkable. Neurological examination was nonfocal.

**Figure 1:**
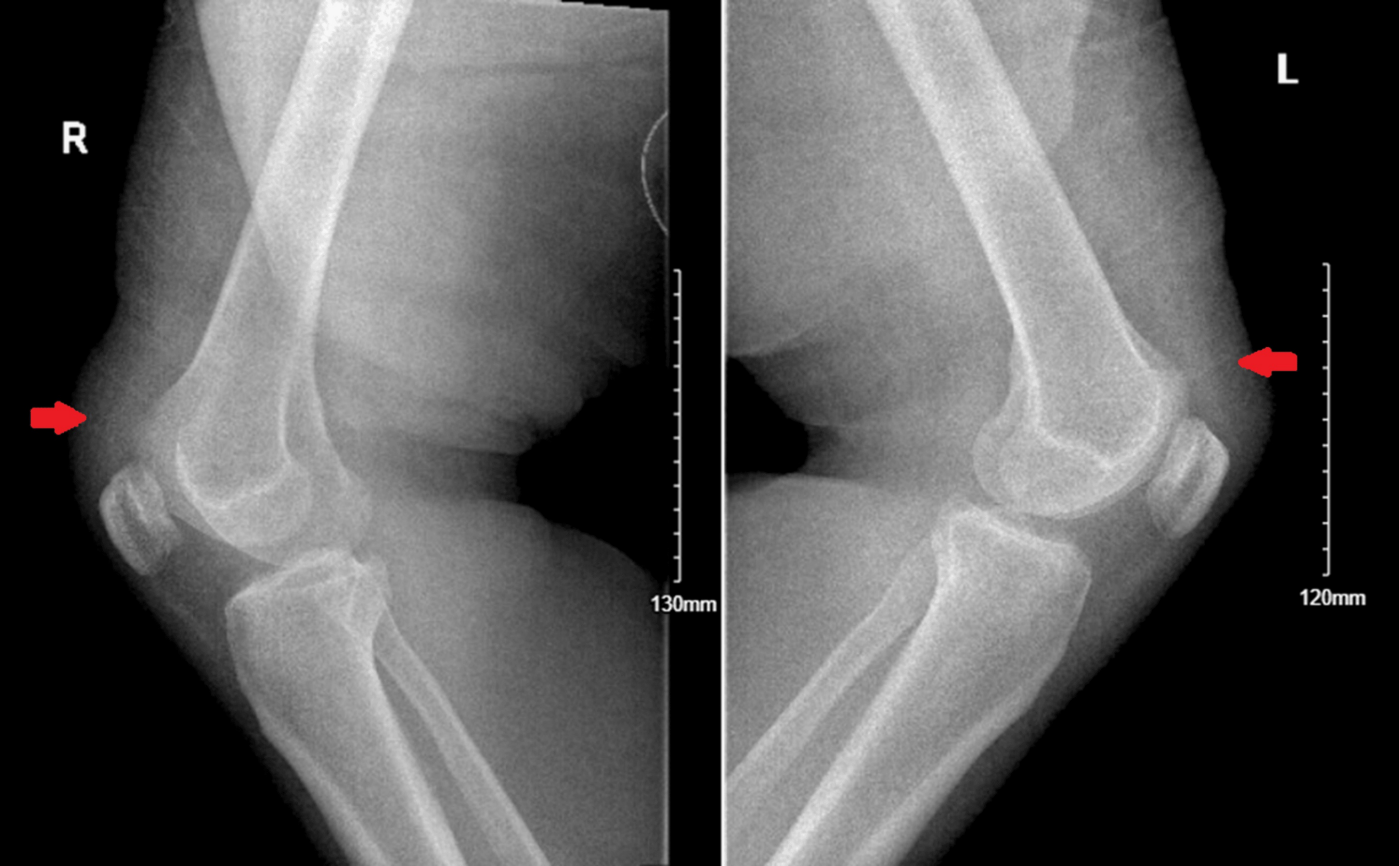
X-ray of bilateral knees in lateral view demonstrating small suprapatellar effusions

**Figure 2:**
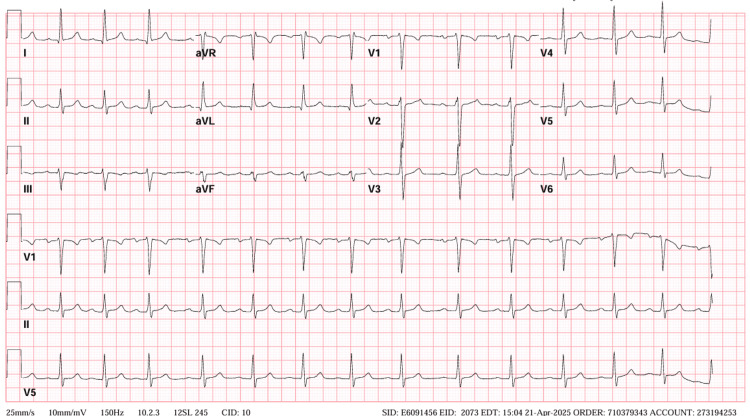
ECG showing prolonged PR interval (208 ms)

Knee X-rays showed bilateral small suprapatellar effusions (Figure 1). Electrocardiogram (ECG) revealed sinus rhythm with a prolonged PR interval of 208 ms (Figure 2). The prolonged PR interval was new compared with a previous ECG a year ago. Complete blood count showed hemoglobin of 10.9 g/dL, WBC count of 9.93 x 10⁹/L, and platelet count of 302 x 10⁹/L. Erythrocyte sedimentation rate (ESR) was 101 mm/hr, and C-reactive protein (CRP) was 227 mg/L. Rheumatoid factor and anti-cyclic citrullinated peptide (anti-CCP) antibodies were negative, as well as the antinuclear antibody (ANA) panel. Tick-borne panel and Lyme IgG/IgM immunoblot were negative. The antistreptolysin O (ASO) titer was elevated at 1,600 IU/mL. Renal and liver function tests were within normal limits. Synovial fluid analysis of the right knee showed a WBC count of 5,000 cells/mm³ (70% polymorphonuclear leukocytes (PMNs)), negative Gram stain, and no crystals (Table 1). Blood cultures and synovial fluid cultures were negative.

**Table 1:** Pertinent laboratory investigations PMN: polymorphonuclear leukocytes

Parameter	Patient value	Normal/reference range
Erythrocyte sedimentation rate	101 mm/hr	0-15 mm/hr
C-reactive protein	227 mg/L	0-5 mg/L
Rheumatoid factor	Negative	Negative
Anti-cyclic citrullinated peptide	Negative	Negative
Antinuclear antibody	Negative	Negative
Antistreptolysin O titer	1,600 IU/mL	<200 IU/mL
Synovial fluid		
WBC	5,000 cells/mm³ (70% PMN)	<2000 cells/mm³
Gram stain	Negative	Negative
Crystals	Negative	Negative

**Figure 3:**
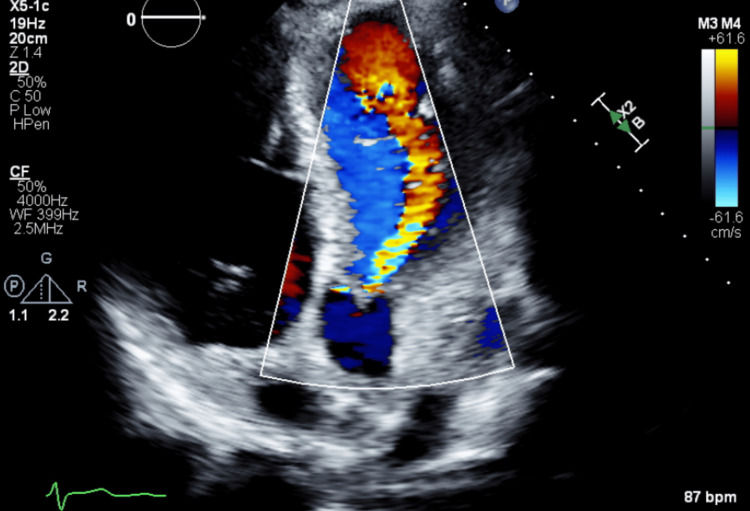
Echocardiography in apical five-chamber view with Doppler demonstrating mild aortic regurgitation

Based on one major (polyarthritis) and three minor criteria (fever, prolonged PR interval, elevated ESR or CRP), along with evidence of a recent GAS infection (elevated ASO titer), the diagnosis of ARF was established according to the 2015 revised Jones criteria [5]. Echocardiography revealed mild aortic regurgitation (AR) and trace mitral regurgitation (MR), with a left ventricular ejection fraction of 55% (Figure 3).

The patient was treated with naproxen 500 mg twice daily, with significant improvement in joint pain. He received a single dose of intramuscular penicillin G (1.2 million units) for eradication therapy and was started on azithromycin 250 mg daily for secondary prophylaxis. Unfortunately, he was lost to follow-up after discharge.

## Discussion

The initial differential diagnosis in this case was broad and included late manifestations of Lyme disease, gout, initial presentation of rheumatoid arthritis, other autoimmune conditions, and septic arthritis. Lyme disease was considered due to the presence of arthritis and a prolonged PR interval, as well as a prior history of presumed Lyme disease. However, review of records indicated that the earlier diagnosis was based solely on a sixth nerve palsy and positive Lyme serology without further workup. The patient had received treatment with doxycycline for 14 days at that time. Current Lyme serologies were negative. Thus, Lyme disease was ruled out.

Synovial fluid analysis ruled out septic arthritis and crystal arthropathy. Rheumatoid arthritis was considered unlikely due to the acute onset, migratory pattern, and asymmetric joint involvement. Laboratory tests for rheumatoid arthritis and systemic lupus erythematosus were negative. Infective endocarditis was deemed unlikely, given negative blood cultures and the absence of vegetations on echocardiography.

The elevated ASO titer raised suspicion for adult-onset ARF. There was no history of ARF in childhood and no recent sore throat prior to presentation. However, given the presence of one major criterion (polyarthritis) and two minor criteria (prolonged PR interval and elevated ESR and CRP), along with evidence of preceding GAS infection (elevated ASO titer), the diagnosis of ARF was established.

An initial attack of ARF in adults is more challenging to diagnose than in children, given the broader differential diagnosis of arthritis in this population. Furthermore, the incidence of rheumatic fever has declined dramatically over the past half-century in the US [6]. As a result, many clinicians have limited or no clinical experience with this condition, making recognition and diagnosis more difficult. ARF is traditionally considered a pediatric disease, and episodes in adults typically represent a recurrence of disease acquired during childhood [1]. Although initial attacks in adulthood are rare, sporadic outbreaks have been reported, such as among military recruits in the US in 1987 [7].

The clinical features of adult-onset ARF have not been extensively studied. However, one study reviewing 23 adult patients with ARF found that arthritis and carditis were the only two major Jones criteria commonly observed in adult cases [8]. Nevertheless, case reports exist of adult ARF presenting with other major manifestations, such as Sydenham’s chorea [9].

Some patients may develop isolated arthritis following a recent GAS infection without fulfilling the diagnostic criteria for ARF. These cases are classified under a distinct entity known as post-streptococcal reactive arthritis (PSRA) [10]. In PSRA, arthritis typically develops within ten days of infection, as opposed to the latency period of two to three weeks seen in ARF. Importantly, despite the potential for cardiac involvement in children with PSRA, adult patients with PSRA are not at increased risk of developing valvular heart disease [10].

In a study that examined eight adults aged 18 years or older with initial attacks of ARF, all presented with high-grade fever and pharyngitis, followed by migratory polyarthritis with a latency period ranging from one to four weeks [11]. Interestingly, our patient did not report a recent history of pharyngitis. One possible explanation could be recall bias. However, a study demonstrated that recollection of sore throat was significantly higher in older children and young adults compared with younger children [12]. A more likely explanation is that the GAS infection may have been subclinical, producing no noticeable symptoms. Thus, even in the absence of reported pharyngitis, evidence of preceding GAS infection should be sought using ASO or antideoxyribonuclease B (ADB) antibody titers, throat culture, or rapid antigen detection testing. Among these, antibody testing is likely the most informative due to the delayed onset of ARF following GAS infection. Throat cultures are typically negative in approximately 75% of cases by the time ARF symptoms manifest [13].

The cardiac sequelae of initial adult-onset ARF remain unclear, with conflicting data reported in the literature. During the 1987 outbreak among US military recruits, three patients developed persistent MR [7]. Conversely, in a separate study involving 53 patients, although eight presented with carditis, the cardiac involvement was mild and transient [14]. In our case, the patient demonstrated a prolonged PR interval on ECG, and echocardiography revealed mild AR and trace MR, findings suggestive of subclinical carditis. There was no clinically appreciable murmur or signs of heart failure. Unfortunately, the patient was lost to follow-up, and repeat interval echocardiographic assessment could not be performed. The implications of loss to follow-up can be significant. Patients with rheumatic MR require follow-up echocardiography every 6-12 months, depending on the severity of valvular regurgitation, to monitor for progression and to guide timely interventions such as valve repair or replacement.

Treatment of adult ARF does not differ from that of childhood ARF. The goals of treatment consist of the eradication of GAS infection, whether or not symptoms of ongoing infection are present. This is typically done using penicillin G intramuscularly. Arthritis typically has a good response to NSAIDs, as in our case. Chronic antimicrobial prophylaxis is recommended to prevent recurrent attacks of ARF, which may lead to the development or progression of RHD.

## Conclusions

This case underscores the diagnostic challenges of ARF in adults, particularly in those without a prior attack of ARF or preceding pharyngitis. Clinicians should consider ARF in the differential diagnosis of acute polyarthritis, especially when no other apparent cause is identified. The presence of extra-articular features, such as carditis, should raise clinical suspicion. Although the incidence of ARF in the US has declined, the disease has not been eradicated. Prompt recognition and treatment of ARF are crucial to prevent long-term cardiac sequelae. Secondary prophylaxis with long-term antibiotics plays a central role in preventing subsequent episodes of GAS infection, which in turn reduces the development or progression of RHD. Furthermore, echocardiographic monitoring is essential in patients with carditis to evaluate for disease progression and guide timely interventions such as valve replacement or repair. Additional research on adult ARF is needed to guide evidence-based management strategies.
